# Functional convergence of *gliP* and *aspf1* in *Aspergillus fumigatus* pathogenicity

**DOI:** 10.1080/21505594.2018.1482182

**Published:** 2018-07-27

**Authors:** Hong Liu, Wenjie Xu, Norma V. Solis, Carol Woolford, Aaron P. Mitchell, Scott G. Filler

**Affiliations:** a Division of Infectious Diseases, Los Angeles Biomedical Research Institute at Harbor-UCLA Medical Center, Torrance, CA, USA; b Department of Biological Sciences, Carnegie Mellon University, Pittsburgh, PA, USA; c Department of Medicine, David Geffen School of Medicine at UCLA, Los Angeles, CA, USA

**Keywords:** Aspergillus fumigatus, virulence, gliotoxin, host response, mycotoxin

## Abstract

Gliotoxin contributes to the virulence of the fungus *Aspergillus fumigatus* in non-neutropenic mice that are immunosuppressed with corticosteroids. To investigate how the absence of gliotoxin affects both the fungus and the host, we used a nanoString nCounter to analyze their transcriptional responses during pulmonary infection of a non-neutropenic host with a gliotoxin-deficient Δ*gliP* mutant. We found that the Δ*gliP* mutation led to increased expression of *aspf1*, which specifies a secreted ribotoxin. Prior studies have shown that *aspf1*, like *gliP*, is not required for virulence in a neutropenic infection model, but its role in a non-neutropenic infection model has not been fully investigated. To investigate the functional significance of this up-regulation of *aspf1*, a Δ*aspf1* single mutant and a Δ*aspf1* Δ*gliP* double mutant were constructed. Both Δ*aspf1* and Δ*gliP* single mutants had reduced lethality in non-neutropenic mice, and a Δ*aspf1* Δ*gliP* double mutant had a greater reduction in lethality than either single mutant. Analysis of mice infected with these mutants indicated that the presence of *gliP* is associated with massive apoptosis of leukocytes at the foci of infection and inhibition of chemokine production. Also, the combination of *gliP* and *aspf1* is associated with suppression of CXCL1 chemokine expression. Thus, *aspf1* contributes to *A. fumigatus* pathogenicity in non-neutropenic mice and its up-regulation in the Δ*gliP* mutant may partially compensate for the absence of gliotoxin.

**Abbreviations**:PAS: periodic acid-Schiff; PBS: phosphate buffered saline; ROS: reactive oxygen species; TUNEL: terminal deoxynucleotidyl transferase dUTP nick-end labeling

## Introduction

*Aspergillus fumigatus* is the most common cause of invasive pulmonary aspergillosis. This fungus expresses a variety of virulence factors that enable it to adhere to and invade host cells and to survive within the hypoxic, nutrient-limited environment of the lung [1–9]. Furthermore, *A. fumigatus* produces a variety of secondary metabolites that may alter the function of host cells and contribute to virulence. Indeed, the *A. fumigatus* genome contains approximately 34 secondary metabolite gene clusters, of which only a subset have been functionally characterized [10]. Perhaps the best-studied secondary metabolite of *A. fumigatus* is gliotoxin. This toxin has multiple effects on the host, including damaging respiratory epithelium, inhibiting the NF-κB transcription factor, altering neutrophil production of reactive oxygen species (ROS), inhibiting macrophage phagocytosis, and inducing apoptosis of monocytes and dendritic cells [11–15]. Gliotoxin is necessary for maximal virulence in experimental mouse models of invasive pulmonary aspergillosis [16,17]. Furthermore, it is detectable in the serum of patients with invasive aspergillosis, suggesting that it also contributes to the pathogenesis of this disease in humans [18].

GliP catalyzes the formation of the diketopiperazine scaffold, which is the first step in the synthesis of gliotoxin, and *A. fumigatus* mutants with deletion of *gliP* have complete absence of gliotoxin production [16,17,19–21]. While Δ*gliP* mutants retain wild-type virulence in leukopenic mice, they have reduced virulence in non-neutropenic mice that have been immunosuppressed with corticosteroids, suggesting that the main role of gliotoxin in virulence is to disrupt leukocyte function.

The infection environment is a unique niche in which host-pathogen interplay leads to the emergent property of virulence [22]. Distinct features of the infection environment have been revealed by diverse approaches that include histological analysis [23,24], imaging mass spectrometry [25], and transcriptional profiling [26]. We have previously used transcriptional profiling to understand the genetic control of pathogenicity in infected tissue [8,27,28]. We have found that many mutants that are defective in proliferation during infection present alterations of gene expression that can reveal the role of the mutated gene in pathogenicity.

Here we have explored the role of *gliP* in *A. fumigatus* virulence through the lens of transcriptional profiling of both the fungus and the host. We assayed expression of a set of *A. fumigatus* genes that were strategically chosen to reflect a range of environmental stimuli, and a set of host genes that encompass key features of the immune response. Serendipitously, we found that the Δ*gliP* mutant had enhanced expression of the ribotoxin gene *aspf1*. This observation led us to the functional hypothesis that Asp f 1 plays a role in the residual virulence of the Δ*gliP* mutant. Our analysis shows that *aspf1* contributes to *A. fumigatus* pathogenicity in non-neutropenic mice, and suggests that up-regulation of *aspf1* in the Δ*gliP* mutant partially compensates for the absence of gliotoxin.

## Results

### Upregulation of aspf1 in the Δglip mutant during invasive aspergillosis represents a compensatory response

To determine the transcriptional response of *A. fumigatus* during invasive infection, we used a nanoString probeset that contained probes for 97 genes known to function in pathways involved in secondary metabolism, iron acquisition, hypoxia, cell wall stress response, host cell invasion and biofilm formation (Table S1). This set of probes was a custom design. The transcriptional profiling was performed on RNA that was directly isolated from the lungs of mice that had been immunosuppressed with cortisone acetate and then infected with *A. fumigatus* Af293 via an aerosol chamber [29]. This route of inoculation delivered approximately 5 × 10^3^ conidia to the lungs of each mouse. RNA was prepared from whole lung samples after 5 days of infection, approximately one day before the onset of mortality.

We assessed the transcript levels of *A. fumigatus* genes in the wild-type strain and the Δ*gliP* mutant. Uninfected samples yielded 4-fold to > 1000-fold fewer probe counts than infected samples, thus indicating that mouse RNA cross-hybridization did not confound the results (Table S1). The majority of genes in the Δ*gliP* mutant were expressed at wild-type levels in vivo (Table S1). However, relative to the wild-type strain, the Δ*gliP* mutant had increased expression of *zrfA*, which specifies a zinc transporters, and of *aspf2*, which is required for growth under zinc-limited conditions (Table 1) [30,31]. These genes were also significantly up-regulated when the nanoString data were normalized to the *gpdA* housekeeping gene. The increased expression of genes involved in zinc acquisition in the Δ*gliP* mutant suggests that gliotoxin may enhance zinc acquisition by *A. fumigatus*, perhaps by causing death of host cells.

**Table 1.** *A. fumigatus* genes that were differentially expressed in the Δ*gliP* mutant relative to the wild-type strain at 5 days post-infection in the lungs of immunosuppressed mice. These results are based on nanoString probe counts (Table S1). Data are the mean of 3 biological replicates.

Gene	Gene Name	Putative Function	Δ*gliP*/WT
Afu5g02330	*aspf1*	Cell surface – allergen, cytotoxin, upregulated in biofilm and in vivo	6.8
Afu3g12890	*hasA*	Transcription factor – regulates biosynthesis of hexadehydroastechrome	5.1
Afu1g01550	*zrfA*	Zinc transporter – acidic environments	3.8
Afu4g14070		Cell surface – secreted protein, glycosyl transferase	2.3
Afu5g14800		Metabolism – lactate dehydrogenase, downregulated in biofilm	2.2
Afu4g09560	*zrfC*	Zinc transporter – neutral or alkaline environments	2.1
Afu4g09580	*aspf2*	Cell surface – fibrinogen binding protein, required for growth in zinc-limited conditions	2.1
Afu1g14560	*msdS*	Putative 1,2-α-mannosidase; secreted protein; fibrinogen-binding	0.43
Afu6g03540	*acuE*	Metabolism – malate synthase, glyoxylate cycle	0.39
Afu2g12850	*gel3*	Putative GPI anchored β(1–3)glucanosyltransferase	0.34
Afu8g01310		Iron acquisition – metalloreductase	0.15
Afu4g11800	*alp1*	Cell surface – secreted protein alkaline serine protease	0.13
Afu6g09660	*gliP*	Gliotoxin – non-ribosomal peptide synthetase	0.03

Deletion of *gliP* altered the expression of genes involved in cell wall synthesis. There was increased expression of Afu4g14070, which specifies a glycosyl transferase, and reduced expression of *msdS* and *gel3*, which encode a 1,2 α-mannosidase and a β(1,3) glucanosyltransferase, respectively. Collectively, these results suggest that the Δ*gliP* mutant may have an altered cell wall relative to the wild-type strain. This difference may be due either to the direct effects of the *gliP* deletion or to indirect effects caused by an altered host response induced by infection with the Δ*gliP* mutant.

**Figure 1.**
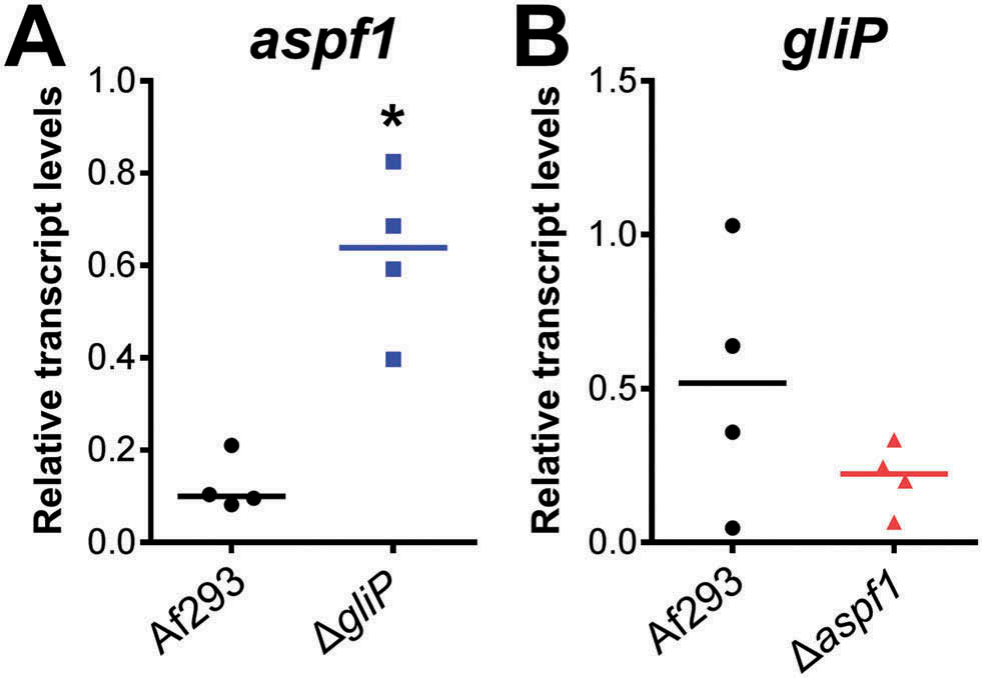
Up-regulation of *aspf1* in the Δ*gliP* mutant during pulmonary infection. Corticosteroid-treated mice were infected via an aerosol with the *A. fumigatus* wild-type (Af293) strain and Δ*gliP* and Δ*aspf1* mutants. After 5 days of infection, the lungs were harvested and total RNA was extracted for real-time PCR analysis. The transcript levels of *aspf1* and *gliP* were normalized to GAPDH. Result are from 4 mice per strain, each tested in triplicate. Horizontal line indicates the median value. *p < 0.02 compared to wild type strain by the Mann-Whitney test.

Relative to the wild-type strain, the Δ*gliP* mutant also had increased expression of genes whose products are involved in the synthesis of toxins. There was upregulation of *hasA*, which specifies a transcription factor that controls production of hexadehydroastechrome [32] and *aspf1*, which encodes a ribotoxin [33],(Table 1). We verified by real-time PCR that *aspf1* transcript levels were increased by approximately 3-fold in mice infected with the Δ*gliP* mutant relative to the wild-type strain (Figure 1(A)). These results indicate that the absence of gliotoxin may induce a compensatory increase in the production of other toxins.

**Figure 2.**
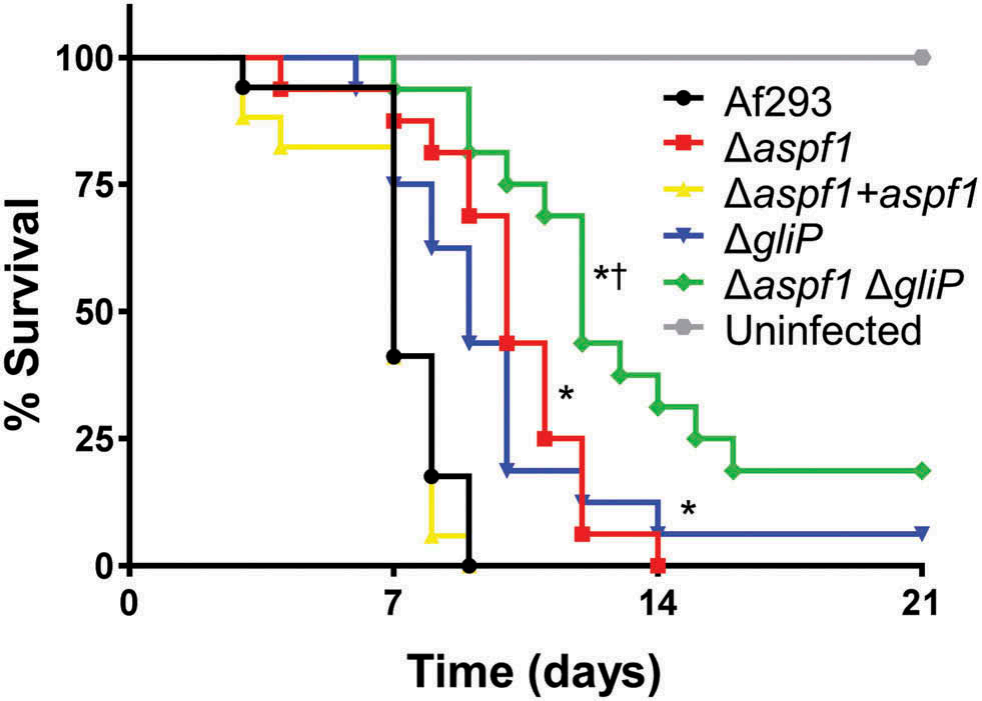
Effects of deletion of *aspf1* and/or *gliP* on the virulence of *A. fumigatus*. Mice were immunosuppressed with cortisone acetate, infected with an aerosol of the indicated strains of *A. fumigatus*, and monitored for survival. Data are the combined results of two independent experiments for a total 16 mice per strain. *P < 0.001 compared to Af293 and the Δ*aspf*1+ *aspf1* complemented strain; †*p* < 0.01 compared to the Δ*glip* and Δ*aspf1* mutants by the log-rank test.

To investigate the relationships among *aspf1, gliP* and virulence, we constructed an *aspf1* deletion in both the wild-type strain and the Δ*gliP* mutant and then tested the virulence of these strains in mice. We found that mice infected with the Δ*aspf1* mutant survived significantly longer than mice infected with either the wild-type strain or the Δ*aspf1+ aspf1* complemented strain (Figure 2). As reported previously [16], mice infected with the Δ*gliP* mutant also lived longer than mice infected with the wild-type strain, surviving similarly to mice infected with the Δ*aspf1* mutant. Strikingly, mice infected with the Δ*aspf1* Δ*gliP* double mutant lived significantly longer than mice infected with either the Δ*aspf1* or Δ*gliP* single mutant. These results suggest that Asp f 1 and gliotoxin make additive contributions to virulence and that the relatively modest virulence defect of the Δ*gliP* mutant is due in part to the compensatory up-regulation of *aspf1*.

To determine if deletion of *aspf1* in turn causes a compensatory up-regulation of *gliP*, we measured the transcript levels of *gliP* in mice infected with the Δ*aspf1* mutant. We found that *gliP* expression was not increased in the Δ*aspf1* mutant (Figure 1(B)). Therefore, while *gliP* has significant impact on expression of *aspf1*, our results indicate that *aspf1* does not significantly influence the expression of *gliP.*

### Both Asp f 1 and gliotoxin contribute to A. fumigatus-induced host cell damage

**Figure 3.**
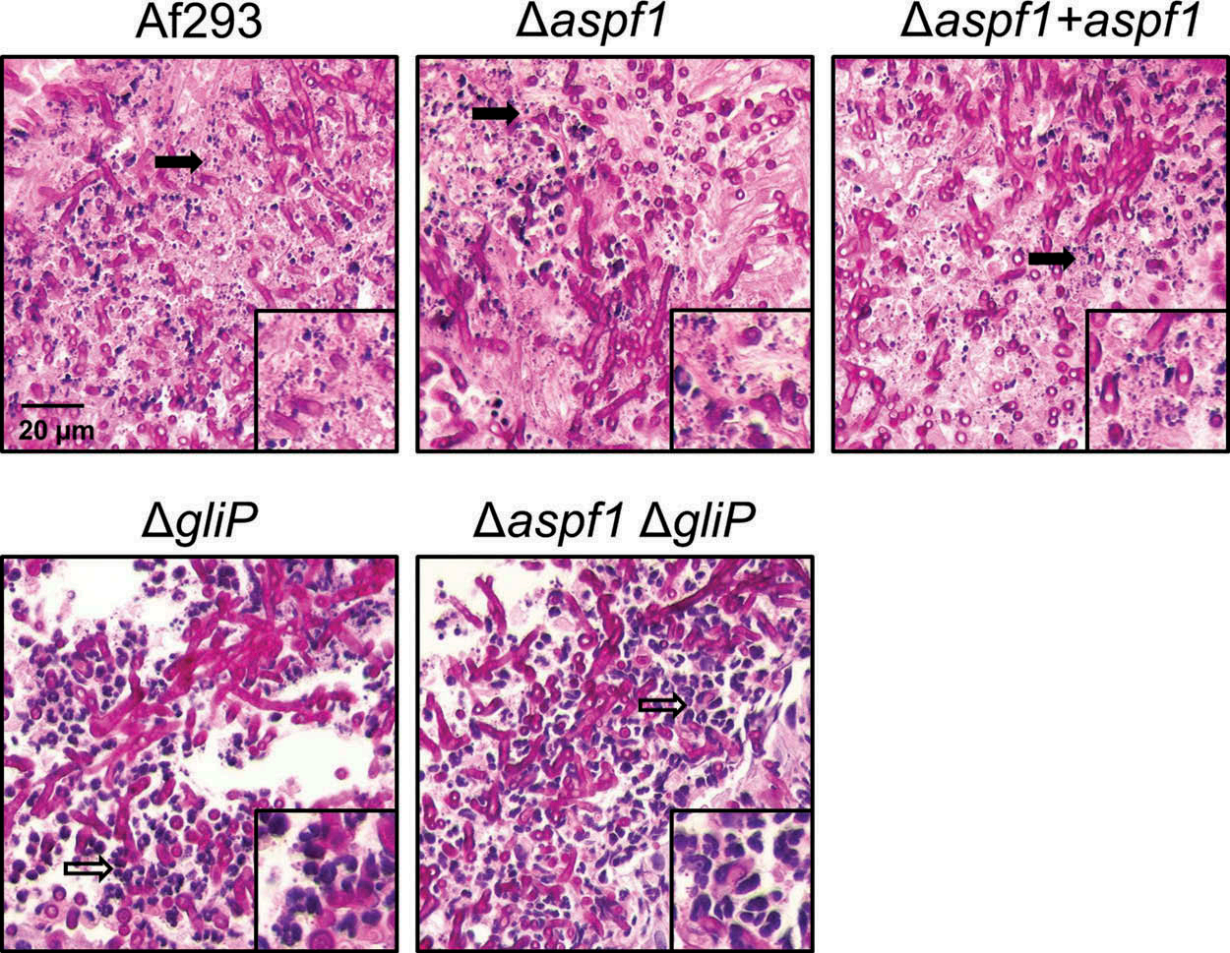
Deletion of *gliP* reduces leukocyte death. Photomicrographs of periodic acid-Schiff (PAS) stained sections of the lungs of mice after 5 days infection with the indicated strains of *A. fumigatus*. Insets show magnified images of the regions indicated by the arrows. Filled arrows indicate leukocyte fragmentation and hollow arrows indicate intact leukocytes. Results are representative of 3 mice per strain. Scale bar, 20 µm.

To understand the function of Asp f 1 in pathogenicity, we carried out histopathologic examination of fungal lesions in the lungs of infected mice. We observed that infection with the wild-type *A. fumigatus* strain caused virtually all of the leukocytes to fragment, leaving almost no intact cells (Figure 3). A similar pattern was observed in the lesions of the mice infected with the Δ*aspf1* mutant and the Δ*aspf1 + aspf1* complemented strain. By contrast, as reported previously [16], fewer fragmented leukocytes and numerous intact neutrophils were present in the fungal lesions of mice infected with the Δ*gliP* mutant. Furthermore, virtually no fragmented leukocytes were seen in the fungal lesions of mice infected with the Δ*aspf1* Δ*gliP* double mutant.

**Figure 4.**
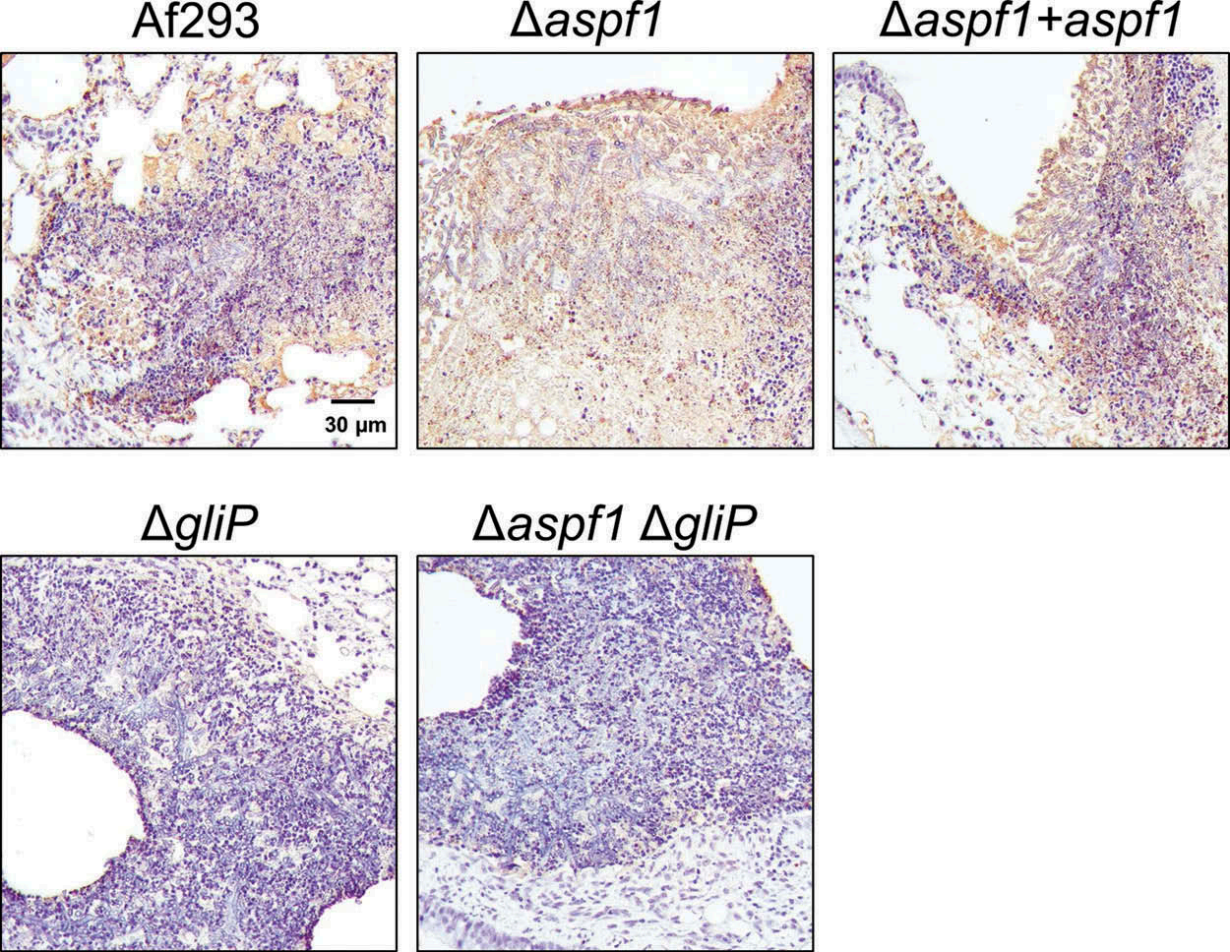
*gliP* is required for *A. fumigatus* to induce leukocyte apoptosis at foci of infection. Photomicrographs of the lungs of mice after 5 days of infection with the indicated *A. fumigatus* strains. Apoptotic cells (brown) were detected by TUNEL staining. Results are representative of 3 mice per strain. Scale bar, 30 µm.

It seemed possible that the leukocyte fragmentation we observed was a result of apoptosis. We assayed apoptosis in vivo through terminal deoxynucleotidyl dUTP nick-end labeling (TUNEL) assays of thin sections of infected lungs. There was extensive leukocyte apoptosis in the fungal lesions of mice infected with the wild-type strain, the Δ*aspf1* mutant, and the Δ*aspf1 + aspf1* complemented strain (Figure 4). However, there was much less apoptosis in the lesions of the mice infected with either the Δ*gliP* single mutant or the Δ*aspf1* Δ*gliP* double mutant. These data indicate that the product of *gliP*, either alone or in combination with the product of *aspf1*, induces apoptotic death of leukocytes in foci of *A. fumigatus* infection in mice.

**Figure 5.**
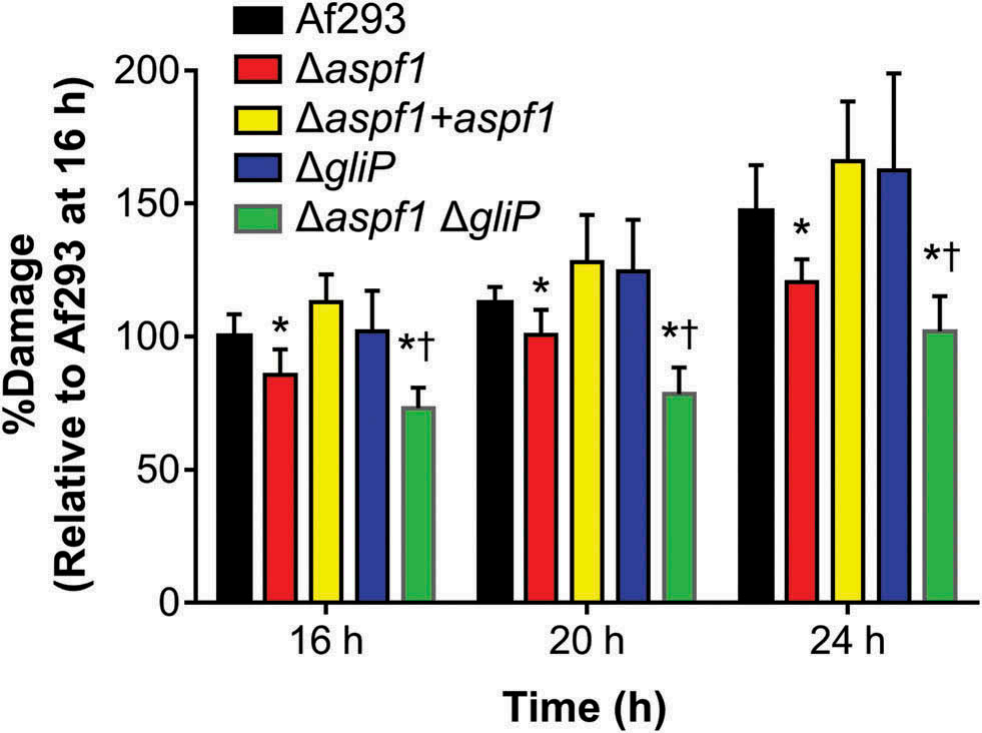
Effects of deletion of *aspf1* and/or *gliP* on the capacity of *A. fumigatus* to damage a mouse macrophage cell line in vitro. The mouse RAW 264.7 macrophage cell line was infected with the indicated strains of *A. fumigatus* for 16–24 h and the extent of host cell damage after 16, 20, and 24 h of infection was measured using a ^51^Cr release assay. Results are mean ± SD of 3 experiments, each performed in triplicate. *P < 0.01 vs Af293 and the Δ*aspf1*+ *aspf1* complemented strain; ^†^*p *< 0.01 vs. the Δ*aspf1* and *gliP* mutants. Statistical significance was analyzed by the student’s t-test with the Holm-Sidak correction for multiple comparisons.

To quantify the capacity of the various *A. fumigatus* mutants to damage leukocytes in vitro, we infected the RAW 264.7 mouse macrophage cell line with these strains and measured the extent of fungal-induced host cell damage using a ^51^Cr release assay. The Δ*aspf1* mutant caused significantly less damage to macrophages than either the wild-type strain or the Δ*aspf1* + *aspf1* complemented strain (Figure 5). Although the Δ*gliP* mutant induced wild-type levels of macrophage damage, the Δ*aspf1* Δ*gliP* double mutant caused less macrophage damage than either the wild-type strain or the Δ*aspf1* mutant. These results indicate that *gliP* and *aspf1* have additive effects on macrophage death.

### Asp f 1 and gliotoxin have additive effects on the host transcriptional response to A. fumigatus

We further assessed the pulmonary response to infection using a 47-gene nanoString probe set for host genes involved in the inflammatory response (Table S2). After 5 days of infection, the host response to the Δ*aspf1* mutant was similar to that induced by the wild-type strain (Tables 2 and S2). By contrast, infection with the Δ*gliP* mutant caused an increase in mRNA levels of CXCL2, CCL3, and CCL4, chemokines that are chemotactic for neutrophils. There was also a significant increase in the mRNA levels of the proinflammatory cytokine TNF-α. In the lungs of mice infected with the Δ*aspf1* Δ*gliP* double mutant, there was less upregulation of CXCL2, CCL3, CCL4, and TNF-α (Tables 2 and S2).

**Table 2.** Expression ratios of host genes that were differentially expressed in mice infected for 5 days with the indicated *A. fumigatus* mutants. These results are based on nanoString probe counts (Table S2). Data are the mean of 4–5 biological replicates.

Host Gene	Gene Product	Δ*aspf1*/WT	Δ*gliP*/WT	Δ*aspf1* Δ*glip*/WT
CXCL2	Macrophage inflammatory protein 2α	0.8	5.8	2.2
CCL4	Macrophage inflammatory protein 1β	0.9	5.7	3.5
CCL3	Macrophage inflammatory protein 1α	0.9	4.9	3.0
TNF	Tumor necrosis factor-α	0.9	3.0	1.4

**Figure 6.**
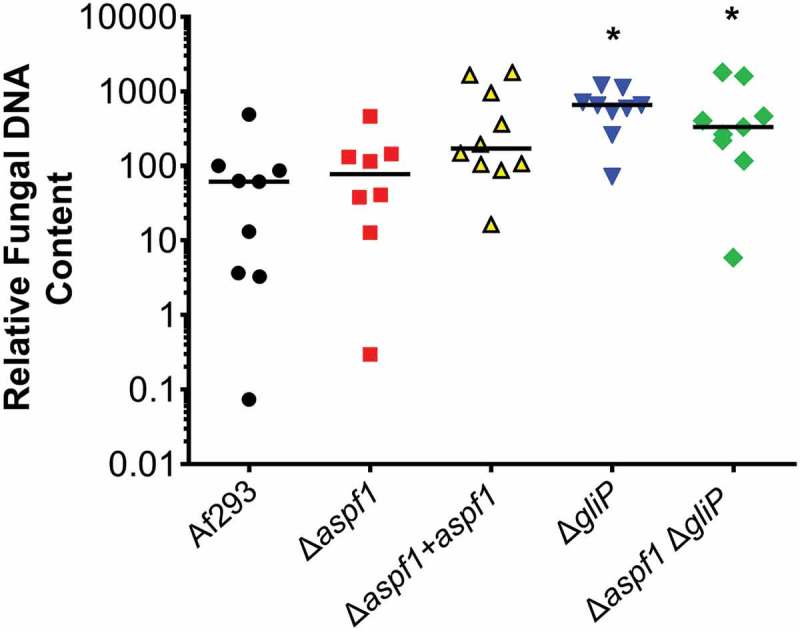
Effects of deletion of *aspf1* and/or *gliP* on the pulmonary inflammatory response. Corticosteroid-treated mice were infected with the indicated strains for 5 days, after which the mice were sacrificed and the lungs were harvested for RNA extraction. The transcript levels of the indicated chemokine and cytokine genes were determined by real-time PCR. Each symbol indicates the result from an individual mouse and the horizontal lines indicate the median values. *P < 0.03 vs Af293; ^†^*p *< 0.03 vs. the *gliP* mutant by the Mann-Whitney test.

To verify and extend these results, we used real-time PCR to determine the transcript levels of 7 genes in the lungs of mice infected with the various *A. fumigatus* strains. Consistent with the nanoString results, we found that infection with the Δ*aspf1* mutant induced the same transcriptional response as the wild-type strain (Figure 6). Infection with the Δ*gliP* mutant significantly stimulated the expression of CXCL2, CCL3, CCL4, CCL7, TNF-α, and IL-6, whereas infection with the Δ*aspf1* Δ*gliP* double mutant significantly decreased the expression of CXCL2, CCL7, TNF-α, and IL-6. Also, infection with the double mutant actually suppressed the transcript levels of CXCL1 to below levels induced by the wild-type strain. Overall, these results suggest during invasive aspergillosis, the absence of *gliP* results in a significant increase in the transcript levels of multiple chemokines and pro-inflammatory cytokines. By contrast the combined absence of *gliP* and *aspf1* results in a weaker inflammatory response.

### Pulmonary fungal burden does not correlate with survival in mice infected with toxin-deficient mutants

**Figure 7.**
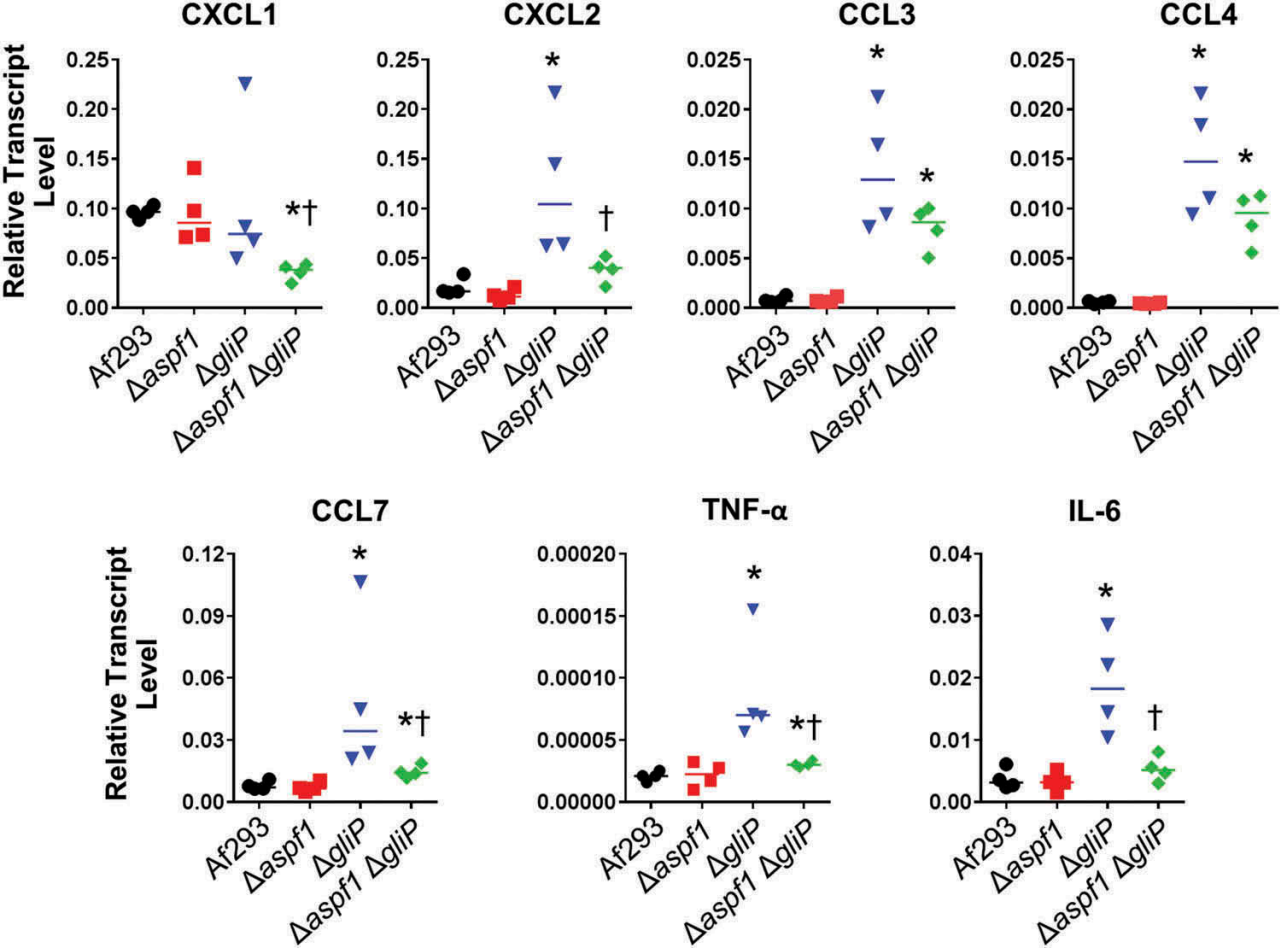
Deletion of *gliP* results in increased pulmonary fungal burden. Mice were infected with the indicated strains of *A. fumigatus* and their pulmonary fungal burden was determined at day 5 by quantitative PCR. Results are from a single experiment using 8–10 mice per strain. Each symbol represents the fungal burden of an individual mouse. Horizontal lines indicate the median value. *P < 0.02 vs Af293 by the Mann-Whitney test.

To assist with interpretation of the inflammatory response data, we used quantitative PCR to measure the pulmonary fungal burden of mice infected with the various *A. fumigatus* strains. After 5 days of infection, mice infected with the wild-type strain, the Δ*aspf1* mutant, and the Δ*aspf1*+ *aspf1* complemented strain had similar fungal burdens, while mice infected with the Δ*gliP* mutant and the Δ*aspf1* Δ*gliP* double mutant had significantly higher fungal burden than mice infected with the wild-type strain (Figure 7). Thus, the attenuated virulence of the mutants was due to an alteration in the host inflammatory response induced by the absence of *gliP* and *aspf1* and was not due to impaired capacity of the mutants to proliferate within the host.

## Discussion

Gliotoxin has multiple negative effects on the host, and it is surprising that gliotoxin-deficient mutants have a relatively modest virulence defect [16,17]. Our profiling data presented here provide an explanation for this unexpected result. Specifically, we observed that the absence of *gliP* led to up-regulation of *aspf1*. Functional analysis of single and double mutant strains is consistent with our understanding that Asp f 1 has a distinct mechanism of action from gliotoxin. However, Asp f 1 and gliotoxin have additive effects on the host, and thus converge to promote pathogenicity.

The finding that *aspf1* transcript levels were increased in the Δ*gliP* mutant suggests that there is a feedback mechanism whereby reduced levels of gliotoxin lead to increased production of Asp f 1. By contrast, Asp f 1 does not appear to regulate the production of gliotoxin because *gliP* transcript levels were unchanged in the Δ*aspf1* mutant. Our hypothesis is that *gliP* affects *aspf1* transcript levels indirectly, perhaps through the liberation of host cell nutrients that results from gliotoxin-mediated apoptosis.

Asp f 1 is a ribotoxin [33] that shares sequence homology with α-sarcin, restrictocin, and mitogillin. It functions by penetrating the cell membrane and cleaving a single phosphodiester bond of the 60S rRNA, which halts protein synthesis [34]. We found that mice infected with the Δ*aspf1* mutant had prolonged survival, implicating Asp f 1 in *A. fumigatus* virulence. This finding contrasts with a previous report that deletion of *aspf1* has no effect on virulence [35]. However, a notable difference between our study and the previous one was that we tested the virulence of the Δ*aspf1* mutant in non-neutropenic mice, whereas the previous study used leukopenic mice. If leukocytes are the principal target of Asp f 1, then this toxin would be unlikely to influence virulence in leukopenic animals.

The greatly reduced apoptosis in the fungal lesions in mice infected with the Δ*gliP* and Δ*aspf1* Δ*gliP* mutants suggests that gliotoxin is a major cause of leukocyte apoptosis in corticosteroid-treated mice. We observed that the inflammatory infiltrate that accumulated in foci of *A. fumigatus* infection in the lung was composed of a mixture neutrophils and mononuclear cells. In vitro studies indicate that while gliotoxin causes apoptosis of monocytes, it does not cause apoptosis of neutrophils [11]. However, we observed that when mice were infected with strains of *A. fumigatus* that contained a functional copy of *gliP*, virtually all leukocytes within the foci of infection had undergone apoptosis. These results suggest that gliotoxin is capable of causing neutrophil apoptosis in vivo.

During infection in mice, we were unable to detect any effect of deletion of *aspf1* on leukocyte fragmentation or apoptosis. We speculate that the strong induction of apoptosis by gliotoxin masked any additional effect of Asp f 1 on leukocyte apoptosis in vivo. However, our in vitro studies showed that Δ*aspf1* mutant had reduced capacity to damage the RAW 264.7 mouse macrophage cell line, even though the Δ*gliP* mutant caused wild-type levels of damage to these cells. Also, the Δ*aspf1* Δ*gliP* double mutant caused less damage that either single mutant. These results suggest that in vitro, Asp f 1 causes host cell damage, which masks the effects of gliotoxin; the capacity of gliotoxin to damage these cells is only apparent in the absence of Asp f 1.

By nanoString and real-time PCR profiling of the host response to the various mutants, we found that infection with the Δ*gliP* mutant enhanced expression of multiple chemokines, including CXCL2, CCL3, CCL4, and CCL7, which recruit neutrophils and macrophages to sites of inflammation. These results suggest that one function of gliotoxin is to inhibit leukocyte recruitment. As many of these chemokines are synthesized by macrophages, we speculate that their increased expression in mice infected with the Δ*gliP* mutants was due to reduced death of macrophages in the fungal lesions. In mice infected with the Δ*gliP* mutant, there was also elevated expression of IL-6 and TNF-α, suggesting that gliotoxin also inhibits the production of these cytokines, both of which are required for the host defense against invasive aspergillosis [36–38].

Analysis of the response of mice to infection with the Δ*aspf1* Δ*gliP* double mutant suggested that the absence of both Asp f 1 and gliotoxin results in a weaker inflammatory response relative to mice infected with the Δ*gliP* single mutant. In mice infected with the double mutant, there were reduced mRNA levels of multiple chemokines, as well as IL-6 and TNF-α. These results suggest that in the absence of gliotoxin, Asp f 1 may induce a proinflammatory response. The prolonged survival of mice infected with the Δ*aspf1* Δ*gliP* double mutant suggests that both gliotoxin and Asp f 1 enhance the extent of pulmonary disease caused by *A. fumigatus*.

A notable finding was that mice infected with either the Δ*gliP* single mutant or the Δ*aspf1* Δ*gliP* double mutant had markedly increased pulmonary fungal burden relative to mice infected with the wild-type strain. Previously, we had found that mice infected with a Δ*gliP* mutant had slightly, but significantly greater pulmonary fungal burden than mice infected with the wild-type strain [16]. However, in our previous study, the pulmonary fungal burden was determined by quantitative culture, which is much less sensitive than quantitative PCR [39]. The current data demonstrate that when investigating toxin-deficient mutants of *A. fumigatus*, pulmonary fungal burden may be dissociated from mortality.

It seems paradoxical that although mice infected with either the Δ*gliP* mutant or the Δ*aspf1* Δ*gliP* double mutant had a stronger proinflammatory response than mice infected with the wild-type strain, they had a higher pulmonary fungal burden. One potential explanation for this result is that gliotoxin has been reported to increase the production of reactive oxygen species (ROS) in neutrophils that have been exposed to corticosteroids [11]. We speculate that this increased ROS production may represent a non-protective inflammatory response. Although it may enhance neutrophil killing of *A. fumigatus* in vivo, it may also increase damage of the surrounding tissue, leading to accelerated mortality of the infected mice. Based on this model, there is less ROS production by neutrophils in mice infected with gliotoxin-deficient mutants, which results in less fungal killing, but also reduced tissue damage and therefore delayed mortality. This model is consistent with the findings of Balloy et al., who determined that in corticosteroid-treated mice with invasive pulmonary aspergillosis, mortality is due in part to dysregulation of the host inflammatory response to the fungus [40].

Collectively, our results indicate that deletion of *gliP* leads to the compensatory up-regulation of *aspf1*, which largely maintains *A. fumigatus* pathogenicity. Our data add further support to the concept that secondary metabolites produced by *A. fumigatus* cause a dysregulation of the host inflammatory response that contributes to lethality even as it limits fungal growth. In addition to producing gliotoxin and Asp f 1, *A. fumigatus* synthesizes multiple additional secondary metabolites, including neosartoricin, fumagillin, pseurotin, trypacidin, and questin [41–44]. Based on the current data, the inter-relationships among these toxins, in terms of their roles in virulence and expression in different microniches within the host, are likely to be quite complex.

## Methods

### Strains, media and growth conditions

*A. fumigatus* Af293 (a generous gift from P. Magee) was used as the wild-type parent strain in all experiments [45]. A list of all strains used in the experiments and their relevant genotypes is provided in Table 3. All *A. fumigatus* strains were grown on Sabouraud dextrose agar (Difco) at 37°C for 7 d prior to use. Conidia were harvest with phosphate-buffered saline (PBS) containing 0.1% Tween 80 (Sigma-Aldrich) and enumerated with a heamacytometer.

**Table 3.** Strains of *A. fumigatus* used in the experiments.

Strain	Genotype	Reference
Af293	Wild-type	[45]
Δ*aspf1*	*Aspf1::hph*	This study
Δ*aspf1*+ *aspf1*	*Aspf1::hph* p*aspf1-ble*	This study
Δ*gliP*	*gliP::hph*	
Δ*aspf1*+ Δ*gliP*	*Aspf1::ble gliP::hph*	This study

### Mutant construction

A split marker strategy was used to disrupt the *aspf1* (Afu5g02330) protein coding sequence [46]. A DNA fragment containing 1352 bp of the 5ʹ-flanking sequence of *aspf1*was PCR-amplified from Af293 genomic DNA using primers Aspf1F4 and Aspf1F3 (Table S3). The resulting product was cloned into plasmid pNLC106 at the KpnI-XbaI sites [46]. Using this plasmid as the template, a DNA fragment containing the *aspf1* 5ʹ-flanking region fused to the 5ʹ portion of *hph* was amplified by PCR using primers Aspf1F4 and HY (Table S3). Next, 1413 bp of 3ʹ-flanking sequence of *aspf1* was amplified using primers Aspf1F2 and Aspf1F1 (Table S3), and a 3ʹ fragment of *hph* was amplified from plasmid pAN7 [47] using primers HYG-F and HYG-R (Table S3). A fragment of the *aspf1* 3ʹ-flanking region linked to the 3ʹ portion of *hph* was obtained by fusion PCR using primers Aspf1F1 and YG (Table S3). Finally *A. fumigatus* Af293 was transformed with both fragments by spheroplasting, after which hygromycin resistant clones were screened for deletion of *aspf1* by colony PCR using primers Aspf1-RT-F and Aspf1-RT-R (Table S3).

To construct the Δ*aspf1* Δ*gliP* double mutant, 1352 bp of the *aspf1* 5ʹ flanking sequence was PCR-amplified from Af293 genomic DNA using primers Aspf1-BLE-F4 and Aspf1-BLE-F3 (Table S3) and 1413 bp of the *aspf1* 3ʹ-flanking sequence was PCR-amplified using primers Aspf1-BLE-F4 and Aspf1-BLE-F3. A DNA fragment containing the phleomycin resistance gene (*ble*) was PCR-amplified from plasmid p402 [48] using primers BLE-F and BLE-R (Table S3). Next, a DNA fragment containing the *aspf1* 5ʹ flanking region linked to the 5ʹ portion of *ble* obtained by fusion PCR using primers Aspf1-BLE-F4 and BL (Table S3). A fragment containing the *aspf1* 3ʹ flanking region linked to the 3ʹ portion of *ble* was obtained similarly using primers Aspf1-BLE-F1 and LE (Table S3). These two fragments were used to transform the *A. fumigatus* Δ*glip* mutant [16]. Phleomycin resistant clones were screened for deletion of *aspf1* by colony PCR using primers Aspf1-RT-F and Aspf1-RT-R.

To complement the Δ*aspf1* mutant, a 3633bp fragment including the intact *aspf1* protein coding sequence was PCR amplified from genomic DNA using primers Aspf1-Com-F and Aspf1-Com-R (Table S3). This fragment was cloned into the NotI-XhoI sites plasmid p402 [48], which was then used to transform the Δ*aspf1* mutant. Phleomycin resistant clones were screened for the presence of the plasmid by colony PCR using primers Aspf1-Com-F and Aspf1-Com-R (Table S3). The transcript level of *aspf1* in the various clones was quantified by real-time RT-PCR using primers Aspf1-RT-F and Aspf1-RT-R (Table S3). The clone in which the *aspf1* mRNA expression was most similar to the wild-type strain was used in all subsequent experiments.

### Mouse model of invasive pulmonary aspergillosis

A non-neutropenic mouse model of invasive aspergillosis was used to assess the transcriptional profile and virulence of the various strains [29]. Briefly, 6 week old, male Balb/c mice (Taconic Laboratories) were immunosuppressed with 7.5 mg cortisone acetate (Sigma-Aldrich) administered subcutaneously every other day starting at day −4 before infection for a total of 5 doses. To prevent bacterial infections, enrofloxacin (Baytril, Western Medical Supply) was added to the drinking water at a final concentration of 0.005% the day before immunosuppression was initiated. The mice were infected by placing them for 1 h in an acrylic chamber into which 12 ml of 1 × 10^9^ conida/ml were aerosolized. Controls mice were immunosuppressed, but not infected.

After 5 days infection, 3 mice infected by each strain were sacrificed and lungs were harvested and saved in liquid nitrogen for RNA extraction. To isolate fungal RNA from the infected mouse lungs, the RNeasy minikit (Qiagen) was used with modifications [8]. Approximately 2.4 ml of buffer RLT with 1% β-mercaptoethanol was added to the lungs from each mouse and the tissue was homogenized in an M tube (Miltenyi Biotec) using a gentleMACS dissociator (Miltenyi Biotec) on setting RNA_02.01. Next, the homogenate was mixed with an equal volume of phenol-chloroform-isoamyl alcohol (25:24:1) and a half volume of zirconium beads (Ambion) and then vortexed with a Mini-Beadbeater (Biospec Products) for 3 min. After centrifugation, the aqueous phase was collected and mixed with an equal volume of 70% ethanol. The RNA was isolated from this mixture using an RNeasy spin column (Qiagen) by following the manufacturer’s instructions.

To assess the virulence of the various *A. fumigatus* strains using survival as the end point, 11 mice were infected with each strain. Shortly after infection, 3 mice from each group were sacrificed, and their lungs were harvested, homogenized and quantitatively cultured to verify conidia delivery to the lung. By this method, the average inoculum was 5 × 10^3^ conidia per mouse for each strain of *A. fumigatus*. The remaining mice were monitored twice daily for survival. The survival experiments were repeated twice and the results were combined.

To measure the pulmonary fungal burden, mice were sacrificed after 5 d of infection, after which their lung were harvested and homogenized in lysis solution (ZR Fungal/Bacterial DNA MiniPrep^TM^, Epigenetics) and DNA was extracted following the manufacture’s protocol. Quantitative PCR was performed using a real-time PCR protocol with *Aspergillus* specific primers ASF1 and ADR1 (Table S3) targeting the 28S rRNA gene [49]. Relative fungal DNA content was quantified by the 2^−ΔΔCT^ method using mouse GAPDH as the reference.

To investigate the integrity and apoptosis of host cells during invasive aspergillosis, the lungs of the mice were harvested after 5 days of infection, fixed in zinc-buffered formalin, and embedded in paraffin. Thin sections were cut and then stained with either periodic acid-Schiff (PAS) or the ApopTag in situ apoptosis detection kit (S7100, EMD Millipore) follow the manufacturer’s directions. After counterstaining the specimens with hematoxylin, they were imaged by bright field microscopy.

### Real-time PCR

The total RNA was reverse transcribed into cDNA using Moloney murine leukemia virus reverse transcriptase (Promega). Real-time PCR was performed using POWER SYBR green PCR master mix (Applied Biosystems) and an ABI 7000 thermocycler (Applied Biosystems). The expression of *A. fumigatus aspf1* and *gliP* was quantified by ΔΔC^t^ method, using *gpdA* as the constitutively expressed gene [9] using the primers listed in Table S3. The expression of mouse CXCL1, CXCL2, CCL3, CCL4, CCL7, IL-6, and TNF-α was also quantified by the ΔΔC^t^ method, using GPDH as the reference gene (Table S3).

### NanoString sample preparation and data analysis

For gene expression profiling of *A. fumigatus*, 10 µg of total RNA isolated from infected mouse lung tissue was mixed with a nanoString codeset mix and incubated at 65°C overnight. The reaction mixtures were loaded into the nanoString nCounter Prep Station for binding and washing, and the resultant cartridge was transferred to the nanoString nCounter digital analyzer for scanning and data collection. A total of 600 fields were captured per sample. The raw data were first adjusted for technical variations in lane-to-lane assay efficiency and background levels as per the nCounter data analysis guidelines. For analysis of *A. fumigatus* gene expression, the adjusted data were then normalized to total probe counts. We also normalized the data to the internal control housekeeping gene *gpdA* to further validate the results. For assessing the host response to infection, 100 ng of total RNA isolated from infected mouse lung tissue was mixed with a nanoString codeset mix and processed as described above. Host response data were normalized using the geometric mean of three internal control genes ACTB, GAPDH and PPIA. All expression ratios were calculated using mean values of three independent biological samples. A gene was considered to be differentially expressed when there was at least a 2-fold difference in the transcript levels and the difference in nanoString counts was statistically significant, as determined by an unpaired, two-tailed student’s t-test (N = 3 for the *A. fumigatus* nanoString; N = 4–5 for the host response nanoString, P ≤ 0.05).

### Cell culture and cell damage assay

The murine RAW 264.7 macrophage cell line (American Type Culture Collection) was grown in Dulbecco’s Modified Eagle’s Medium(American Type Culture Collection) with 10% fetal bovine serum (Gemini Bio-Products), 1% streptomycin and penicillin. The capacity of the various strains to damage the cells was determined using a minor modification of our previously described method [8,50]. RAW cells were grown to confluency in 24-well tissue culture pates and then incubated with ^51^Cr overnight. After rinsing the cells to remove the unincorporated ^51^Cr, they were infected with 5 × 10^5^ germlings of each strain. After 20 h of infection, the medium above the cells was collected and the cells were lysed with 6 N NaOH. The lysed cells were collected with two rinses by RadiacWash (Biodex Medical Systems). The amount of ^51^Cr in the medium and the cell lysate was determined by gamma counting. The spontaneous release of ^51^Cr was determined using uninfected RAW cells that were processed in parallel. The specific release of ^51^Cr was calculated by the following formula: (experimental release-spontaneous release)/(total incorporation-spontaneous release). Each experiment was performed in triplicates and repeated three times.

### Statistical analysis

The data from the in vitro experiments were analyzed by the two-tailed Student’s t-test with the Holm-Sidak correction for multiple comparisons. The survival data were analyzed using the Log-Rank test and the pulmonary fungal burden results were analyzed with the Man-Whitney test. A *P*-value of ≤ 0.05 was considered to be significant.
